# Antibiotic resistance and pathogenicity of Shiga-toxin-producing *Escherichia coli* (STEC) and non-STEC isolated from goats in the Mekong Delta, Vietnam

**DOI:** 10.5455/javar.2025.l908

**Published:** 2025-05-07

**Authors:** Thuan Khanh Nguyen, Trung Thanh Truong, Toan Tri Nguyen, Duy Duc Tran, Thu Thi Chuong Dang, Binh Cong Tran

**Affiliations:** 1 Faculty of Veterinary Medicine, College of Agriculture, Can Tho University, Can Tho City, Vietnam; 2 Faculty of Animal Science, College of Agriculture, Can Tho University, Can Tho City, Vietnam

**Keywords:** Antibiotic resistance, goats, Mekong Delta, STEC, virulent genes.

## Abstract

**Objective:**

Our study is conducted to identify serotypes, antibiotic resistance, heavy metal resistance, and virulent genes in *Escherichia coli* isolated from goats raised in small-scale farms in some provinces of the Mekong Delta, Vietnam.

**Material and Methods:**

A total of 203 *E*. *coli* isolates from goat feces were examined by PCR for serotypes (O8, O9, O25, O26, O45, O103, O146, O157, and O159), eight antibiotic-resistance genes, four heavy-metal-resistance genes, and four pathogenic genes.

**Results:**

By PCR, 20.20% of *E*. *coli* isolates belonging to serotypes O8 (6.40%), O45 (13.30%), and O159 (0.49%) were identified. Antibiotic-resistance genes were recorded at high rates in *E*. *coli* isolates, especially genes *blaampC* (98.52%), *tetA* (50.74%), *sulII* (34.48%), *qnrA* (20.69%), and *aadA1* (20.69%). Moreover, 55.67% of these *E*. *coli* isolates harbored multiple antibiotic-resistance genes. Among heavy-metal-resistance genes, the gene *czcD* encoding for resistance to cobalt, zinc, and cadmium was the most prevalent (59.11%). In addition, the most frequent virulent gene was *stx1* (15.27%), followed by gene *stx2* (6.90%), *eae*, and *hlyA* (1.48%).

**Conclusion:**

These results revealed that goats were a natural reservoir of pathogenic *E*. *coli* serotypes, which could cause severe diseases in animals and humans. Moreover, these *E*. *coli* isolates showed a high ability to resist diverse antibiotics. Thus, managing the prevalence of pathogenic *E*. *coli* is essential for protecting public health in the Mekong Delta.

## Introduction

Livestock can naturally harbor *Escherichia coli*, which causes disease when the host’s immune system is deficient in their gastrointestinal tract [1]. Previous reports indicate *E*. *coli* strains belong to the serotypes producing Shiga toxin and enterotoxin, which are considered typical agents causing diseases in livestock [1–3]. In goats, *E*. *coli* causes diseases at different severities, reducing the growth capacity of goats and causing heavy economic losses for owners [4]. Moreover, Shiga-toxin-producing *E. coli (STEC)* also brings about several ranges of diseases in humans, such as mild diarrhea, hemorrhagic colitis, and hemolytic uremic syndrome with high mortality [5]. A study in the Mid-Atlantic US by Ndegwa et al. [6] identified that STEC isolated from goats belonged to O5, O8, O26, O76, O78, O87, O91, O143, and O146. Moreover, the *hly* and *eae* genes were found in STEC, respectively, and these results indicated that goats harbored STEC belonging to important non-O157 STEC serogroups related to severe human disease.

Additionally, antibiotic resistance in bacteria has become a concern for the veterinary industry, as *E*. *coli* strains harboring antibiotic-resistance genes are frequent. The global emergence of antibiotic-resistant pathogens and commensal bacteria poses significant implications for human and animal health; however, there is a notable lack of published research and surveillance regarding antimicrobial resistance in small ruminants [7]. In Saudi Arabia, it was recorded that antimicrobial-resistant *E*. *coli* strains were detected in goats and transmitted to animals and humans [8]. Research by Ndegwa et al. [9] showed that *E. coli* resisted tetracycline (51.00%) and streptomycin (STEC) and non-STEC isolated from goats in the Mekong Delta, Vietnam. J Adv Vet Anim Res 2025; 12(2):420–426. (30.00%) and indicated genes of *tetB*, *blaTEM*, *aadA*, and *strpA*/*strpB* were detected in 70.00%, 43.00%, 44.00%, and 24.00% of isolated *E*. *coli* from pastured goats resistant to tetracycline, ampicillin, and streptomycin, respectively. In Vietnam, there were a few reports on antibiotic resistance in small ruminants; thus, the study on antibiotic resistance in goats is essential for preventing resistance in treating animals and humans.

Otherwise, heavy metals are present in animal production alongside their clinical application for treating human and animal diseases. At the same time, bacteria have developed various resistance mechanisms to heavy metals via natural selection in response to environments contaminated with heavy metal ions [10]. Moreover, antimicrobial resistance in bacteria is increasingly recognized as a worldwide health issue threatening human and animal health, with the selective pressure from disinfectants and heavy metals significantly contributing to its emergence and dissemination in the food chain [11–13]. Peltier et al. [14] found that subtoxic zinc and antibiotics (ciprofloxacin, oxytetracycline, and tylosin) co-exposure promotes antibiotic resistance via cross-resistance in laboratory-scale wastewater bioreactors, while Berg et al. [15] indicated that copper addition to agricultural soil indirectly promotes resistance to both antibiotics and copper by facilitating horizontal gene transfer in mobile genetic factors.

Moreover, some studies suggest that subinhibitory heavy metal concentrations may increase antibiotic resistance by inducing bacterial mutagenesis [16–18]. Yang et al. [19] indicated that heavy metal-resistance genes were found in *E*. *coli* and *Salmonella* obtained from chicken farms and retail meat, showing a significant correlation with disinfectant and antibiotic-resistance genes, highlighting that their coexistence within plasmids or chromosomes may cause co-resistance to heavy metals, disinfectants, and antibiotics.

In the Mekong Delta, the number of goats has increased because of their adaptive ability to climate change in this region. However, goats were mainly raised in small-scale farms with low hygiene conditions, and the study of pathogens in goats has been limited. Therefore, this study aims to reveal serotypes, antibiotic resistance, heavy metal resistance, and virulent genes in *E*. *coli* isolated from goats raised on small-scale farms. These results are essential for treating and preventing disease transmission in goats and humans.

## Materials and Methods

### Ethical Approval

Feces were collected to isolate *E*. *coli* from goats raised on small-scale farms. The procedure followed the Helsinki Declaration guidelines and the experimental safety principles of Can Tho University, Vietnam. Local government veterinarians observed the sample collection on these farms.

### Determination of E. coli serotypes in goats

From March to June 2024, a total of 203 *E*. *coli* isolates were isolated from meat goats’ feces (hybrid Boer breed) in previous research in a random survey in five small-scale farms, including three in Can Tho City (*n =* 127) and two in Hau Giang province (*n =* 76) in the center of the Mekong Delta, Vietnam. In this study, during the sampling period, goats raised in these farms were divided into two groups: those under 6 months old (*n =* 92) and those 6 months old and older (*n =* 111). The goats were healthy and not treated with antibiotics for at least 1 week.

Each goat’s feces were collected using separate plastic covers placed on the barn floor before being taken to the sampling area; then, they were kept in sterilized plastic bags. In the laboratory, *E*. *coli* was isolated on MacConkey agar (Merck, Germany) and examined through biochemical tests. The *E*. *coli* isolates, which represent each positive sample in each farm, were selected for examination.

The 203 *E*. *coli* isolates were extracted following the boiling method, and the DNA was kept at −20°C for use in this experiment [20]. Nine serotypes of *E*. *coli* isolates were determined. The primer sequences and PCR conditions were applied as in the review article of DebRoy et al. [21] for serotypes O8, O9, O25, O26, O45, O103, O146, O157, and O159.

The PCR reaction (25 μl) includes Mastermix 2X (12.5 μl, Bioline, Canada), forward primer (0.5 μl), reverse primer (0.5 μl), distilled water (9.5 μl), and DNA template (2.0 μl). The purified water without DNA and RNA was the negative control, while the positive controls were *E*. *coli* serotypes detected in cows and beef cattle previously in the Mekong Delta and kept in the Veterinary Food Hygiene Laboratory, Faculty of Veterinary Medicine, College of Agriculture, Can Tho University.

### Determination of antibiotic-resistance and heavy-metal-resistance genes of E. coli isolated from goats

Antibiotic-resistance genes representative of antibiotics/antibiotic groups were detected following the primers and PCR conditions in references, including beta-lactam— *blaampC* [22], *blaTEM* [23]; aminoglycoside*—aadA1*; tetracycline—tetA [24]; colistin—mcr-1 [25]; chloram-phenicol—*cat1* [26]; quinolone—*qnrA* [27]; and sulfon-amide—*sulII* [28].

The PCR primers and conditions were used to detect heavy metal-resistance genes representative of metal resistance following references, including copper–*pcoR*, cobalt, zinc, and cadmium–*czcD*, cobalt and nickel–*cnrA* [14], and silver–*silE* [29].

The PCR mixture and procedure were carried out to determine *E*. *coli* serotypes. Purified water without DNA and RNA was the negative control, while *E*. *coli* isolated from domestic animals in the Mekong Delta harboring these genes were the positive controls.

### Detection of virulent E. coli genes isolated from goats

The PCR primers and conditions were used to detect virulent genes commonly causing severe disease in animals and humans following references, including *stx1* and *stx2* (Shiga toxin), *eae* (intimin), and *hlyA* (hemolysin) [30].

The PCR mixture and procedure were carried out to determine *E. coli* serotypes. Purified water without DNA and RNA was the negative control, while *E. coli* O157 harboring these genes isolated from cattle in the Mekong Delta previously was the positive control.

### Statistical Analysis

The different prevalence of *E*. *coli* serotypes, antibiotic resistance, heavy metal resistance, and virulent genes in goats was determined by the chi-square test. The Pearson chisquare statistic was used at the significance level of 95% in the Minitab 17.0 software (Minitab Pty Ltd, Australia).

## Results

### Prevalence of E. coli serotypes in goats

Three serotypes were identified by PCR from 203 *E*. *coli* isolates, including O8 (6.40%), O45 (13.30%), and O159 (0.49%); however, serotypes O9, O25, O26, O103, O146, and O157 were not detected (Table 1). In this research, these identified serotypes were detected from *E*. *coli* isolated from goats over 6 months old, and the prevalence of these serotypes among small-scale farms showed no significant difference (*p* > 0.05).

**Table 1.** Table 1. Prevalence of *E. coli* serotypes isolated from goats in the Mekong Delta, Vietnam.

Serotypes	Ages of goats		Total (%) (n = 203)	p-value
	< 6 months (%) (n = 92)	= 6 months (%) (n = 111)		
O8	2 (2.17)	11 (9.91)	13 (6.40)	0.025
O45	7 (7.61)	20 (18.02)	27 (13.30)	0.029
O159	0 (0.00)	2 (1.80)	2 (0.49)	< 0.05
Undefined	83 (90.22)	78 (70.27)	161 (79.31)	0.004

### Antibiotic-resistance and heavy-metal-resistance genes of E. coli isolated from goats

Antibiotic-resistant genes were recorded at high rates, especially genes *blaampC* (98.52%), followed by *tetA* (50.74%), *sulII* (34.48%), *qnrA* (20.69%), *aadA1* (20.69%), blaTEM (8.37%), cat1 (5.42%), and mcr-1 (5.42%) in these *E*. *coli* isolates (Table 2). Moreover, 55.67% of these *E*. *coli* isolates harbored multiple drug resistance genes and could harbor from two to six genes examined. The most common gene pattern detected was the *blaampC + tetA + sulII (13.30%)* (Table 3).

**Table 2.** Table 2. The presence of antibiotic genes in *E. coli* isolated from goats (*n =* 203).

Antibiotic/Antibiotic groups	Gene	No. of positive isolates	Percentage (%)
Beta-lactam	blaampC	200	98.52
	blaTEM	17	8.37
Tetracycline	tetA	103	50.74
Sulfonamide	sulII	70	34.48
Quinolone	qnrA	42	20.69
Aminoglycoside	aadA1	42	20.69
Chloramphenicol	cat1	11	5.42
Colistin	mcr-1	11	5.42

**Table 3.** Table 3. Patterns of combined antibiotic-resistance genes in *E. coli* isolated from goats (*n = 203*).

No. of genes	Patterns	No. of isolates	Percentage (%)
2	blaampC + aadA1	9	4.43
	blaampC + sulII	5	2.46
	blaampC + tetA	11	5.42
	blaampC + qnrA	7	3.45
3	blaampC + blaTEM + tetA	4	1.97
	blaampC +blaTEM + aadA1	4	1.97
	blaampC + tetA + qnrA	8	3.94
	blaampC + tetA + sulII	27	13.30
	blaampC + aadA1+ cat1	2	0.99
	blaampC+ aadA1+ tetA	6	2.96
	blaampC + sulII + qnrA	5	2.46
	tetA + sulII + qnrA	4	1.97
4	blaampC + blaTEM + mcr-1+ tetA	2	0.99
	blaampC + aadA1 + cat1 + qnrA	5	2.46
	blaampC + aadA1+ tetA + sulII	5	2.46
	aadA1 + tetA + qnrA + mcr-1	1	0.49
5	blaampC + blaTEM + mcr1+ tetA + sulII	2	0.99
	blaampC + blaTEM + aadA1+ mcr1 + tetA	1	0.49
	blaampC+ blaTEM + aadA1+ tetA+ sulII	2	0.99
	blaampC + cat1 + tetA + sulII + qnrA	2	0.99
6	blaampC + blaTEM + aadA1 + tetA + qnrA + mcr-1	1	0.49
Total		113	55.67

Among heavy-metal-resistance genes, gene *czcD*, which encodes for resistance to cobalt, zinc, and cadmium, was the most prevalent (59.11%), followed by *pcoR* (9.36%) and *silE* (8.37%); however, gene *cnrA* was not detected (Table 4).

**Table 4.** Table 4. The presence of heavy metal resistance genes in *E. coli* isolated from goats (*n =* 203).

Heavy metal resistance	Gene	No. of positive isolates	Percentage (%)
Copper	pcoR	19	9.36
Cobalt-zinc-cadmium	czcD	120	59.11
Cobalt-nickel	cnrA	0	0.00
Silver	silE	17	8.37

### Prevalence of virulent genes of E. coli isolated from goats

In this study, the most frequent virulent gene was *stx1* (15.27%), followed by *stx2* (6.90%), *eae* (1.48%), and *hlyA* (1.48%). Most of these genes were detected in identified *E*. *coli* serotypes O8, O9, and O159 (Table 5). Among those, four *E*. *coli* isolates (O8 = 2, O45 = 2) harbored both genes *stx1* and *stx2* (data not shown).

**Table 5.** Table 5. The presence of virulent genes in *E. coli* serotypes isolated from goats.

Serotypes	No. of examined isolates	Genes			
		stx1 (%)	stx2 (%)	eae (%)	hlyA (%)
O8	13	9 (69.23)^b^	4 (30.77)^a^	1 (7.69)^a^	0 (0.00)
O45	27	9 (33.33)^c^	5 (18.52)^a^	1 (3.70)^a^	2 (7.41)^a^
O159	2	2 (100.00)^a^	0 (0.00)	0 (0.00)	1 (50.00)^a^
Undefined	161	11 (6.83)^d^	5 (3.12)^b^	1 (0.62)^a^	0 (0.00)
Total	203	31 (15.27)	14 (6.90)	3 (1.48)	3 (1.48)

a,b,c,d These letters indicate the significant statistical difference at 95% confidence in each column.

## Discussion

Pathogenic *E*. *coli* is a concern because it can cause animal diseases and be transmitted to humans. This study showed that *E*. *coli* serotypes O8, O45, and O159 were present in goats and detected in goats over 6 months old in the Mekong Delta, Vietnam. Van Hoek et al. [31] commented that the prevalence of STEC carriage among asymptomatic adult animals on farms of dairy goats and sheep was significantly higher than in the entirety of the population, indicating potential zoonotic transmission of STEC. According to Mir et al. [32], the metagenomic analysis suggested that the colonization of STEC is associated with reduced gut microbiota diversity, which improves as the cattle age; therefore, adult cattle become a source of shedding STEC to cattle of other ages. On the other hand, several studies indicated diverse STEC serotypes are present in goats and sheep in different areas, such as O146, O26, O157, and O182 in the Netherlands; O5, O91, O104, O113, and O128 in Iran; O5, O8, O26, O76, O78, O87, O91, O143, and O146 in the Mid-Atlantic US; and O157 in Thailand [6,31,33,34].

In addition, STEC serotypes O26, O45, O103, O111, O121, and O145 are the six most commonly associated with human disease [35,36]. Compared to single strains, the combination of multiple serotypes of *E*. *coli* in the intestine is more likely to lead to disease, and the disease is often more severe in affected animals [37]. Thus, this study revealed that adult goats were considered a natural reservoir of pathogenic *E*. *coli*, especially STEC, and a potential risk to cause diseases for young goats and humans in the Mekong Delta.

Antimicrobial resistance causes challenges in treating diseases in animals and humans and is considered an issue impacting public health. In this study, farmers do not frequently use antibiotics in treating diseases in goats in those small-scale farms; however, the results indicated that these *E*. *coli* isolates harbored several antibiotic-resistance genes, especially genes *blaampC* (98.52%) and *tetA* (50.74%), at significantly high rates. In other studies, Kumar et al. [38] detected genes *blaTEM*, *tetB*, and *tetA* in *E. coli* isolates from sheep and goat feces in India. Moreover, the gene *mcr-1* encodes for colistin resistance in these *E*. *coli* isolates in the Mekong Delta, Vietnam. This antibiotic is one of the “last-line” antibiotics used in humans and requires management in livestock [39,40]. It demonstrated that antibiotic resistance factors were transmitted in goats and husbandry environments in these farms, which could become a risk for public health in this region. A study of *E*. *coli* strains isolated from goat herds in Virginia, US, found that *E*. *coli* strains isolated from this area had a high level of tetracycline resistance, although this antibiotic was never used on the farm [9].

The essential factors that always exist can transfer antibiotic resistance from the intermediate environment to *E*. *coli* strains on livestock, even though farmers did not directly use antibiotics [41]. Furthermore, the antibiotic resistance of *E*. *coli* could not depend so much on resistance genes but requires a combination of many other secondary factors, such as environmental factors, a combination of genes, bacterial adaptation, or antibiotic concentration [25]. Despite frequent antibiotic exposure, antibiotic-resistance genes may not be expressed or show low-level function in some bacteria [42].

On the other hand, multiple antibiotic-resistance genes were found in these *E*. *coli* isolates from goats, showing a risk of multidrug resistance in these isolates. Sharma et al. [43] indicated that *E*. *coli* strains were considered the primary reservoir of antibiotic-resistance genes and caused the transmission of antibiotic-resistance genes in animals, humans, and the environment. At the same time, the diversity and abundance of *E*. *coli* serotypes have helped this species evolve and adapt to the environment. It reported that the multidrug-resistant *E*. *coli* isolated from goat and sheep feces was 51.50% in Al-Madinah, Saudi Arabia [8]. The results underscored sheep and goats as important reservoirs for spreading multidrug-resistant *E*. *coli* and lateral gene transfer of resistance genes. This clearly explains the multi-antibiotic resistance genes of *E*. *coli*, which exchange genes with each other and create multi-resistant strains with diverse phenotypes and genotypes.

Bacteria will develop resistance mechanisms to metals and acquire metal resistance genes [44], and the combined selective pressure of metals and antibiotics is responsible for transmitting resistance genes [45]. Gillieatt and Coleman [46] indicated that the combined selective pressure of heavy metals significantly contributes to the spread and persistence of antibiotic resistance genes in environmental reservoirs. The issue is that antibiotics and metal pollutants often coexist, and their resistance mechanisms share similarities, suggesting a complex evolutionary relationship shaped by this dual pressure. It is well-established that metal resistance genes are genetically associated with antibiotic-resistance genes, facilitated by plasmids, transposons, and integrons that contribute to these resistance elements’ assembly and horizontal transfer. In previous research, the ion Cu2+ interacts with drugs in the body, allowing it to be combined with common antibiotics to increase activity against bacterial growth, limiting drug resistance [47]. Mazhar et al. [48] found that Cd²⁺ and Cu²⁺ were correlated with most antibiotic-resistance genes. This effect may be due to the potent concentration-dependent cytotoxicity of heavy metals such as Zn2+, Ni2+, Cu2+, and Co2+ [49]. Co-resistance occurs between Cu2+ and some antibiotics, such as macrolides commonly used in treatment and glycopeptide antibiotics used as growth promoters in veterinary medicine in previous years [50].

A relevance network analysis indicated a positive correlation between metal-resistance genes, particularly for copper and zinc, and various antibiotic-resistance genes, resulting in a complex interconnected network [51]. Laffite et al. [52] found that Cd²⁺ metal increased the production of genes encoding resistance to the beta-lactam antibiotic group. In China, the presence of a plasmid harboring both the beta-lactam resistance gene (*bla*) and the copper (*pco*) and silver (*sil*) resistance operons was reported in *E*. *coli* isolated from diseased chickens, pigs, and ducks [53]. Furthermore, beyond co-resistance, cross-resistance is likely facilitated by drug efflux systems, modifications in cell membrane structure, outer membrane adaptation, or genetic mutations [54]. Thus, understanding heavy metal resistance in bacteria, including *E*. *coli*, is essential for preventing antibiotic resistance in goats and other animals.

The presence of pathogenic factors in *E*. *coli* reservoirs in adult animals can demonstrate a risk for humans via direct contact or consuming contaminated products. Thus, understanding these factors across diverse sources is essential for informing the development of prevention and control strategies, including biosecurity and vaccination, to safeguard the safety of animal-derived products [55]. Moreover, genotyping STEC is an effective method for elucidating the clonal relationships among various isolates and identifying distinctive genetic patterns linked to specific serogroups or combinations of virulence genes [6]. In this study in the Mekong Delta, STEC (O8, O45, O159) harbored the genes *stx1*, *stx2*, *eae*, and *hlyA*, which indicated these serotypes were virulent and could cause severe diseases in goats and humans through direct or indirect contact.

In the Netherlands, Van Hoek et al. [31] researched STEC isolated from dairy goats and sheep and detected diverse types of genes, *stx1*, *stx2*, and *eae* in these strains. In addition, the close genetic similarity of STEC isolates recovered from both human and small ruminant samples on the same farm suggests the possibility of zoonotic transmission from the animal reservoir to humans within these agricultural environments. In South Africa, virulence genes in STEC isolated from goats were distributed at a high rate, including *stx1*, *stx2*, *eaeA*, and *hlyA* at 60.6%, 72.7%, 22.1%, and 78.0%, respectively. The results indicate that goats in South Africa serve as a reservoir and a potential source of diverse STEC serotypes that pose a virulent hazard to humans [56]. Thus, controlling the prevalence of STEC harboring virulent genes is essential for preventing diseases in goats and humans in the Mekong Delta.

## Conclusion

This study revealed that STEC was frequently found in goats in surveyed small-scale farms in the Mekong Delta. Moreover, these *E*. *coli* isolates possessed a diverse array of significant genes for antibiotic resistance, heavy metal resistance, and virulence. These related agents increased the pathogenicity of *E*. *coli* isolates, especially STEC in goats and humans. Thus, managing the prevalence of these *E*. *coli* serotypes in goats should be implemented and extended to other farm types, alongside evaluating the genetic link between goat and human *E*. *coli* isolates. It helps to assess the epidemiological situation and comprehensively manage *E*. *coli* diseases transmitted between humans and animals.

## Lists of abbreviations

DNA, deoxyribonucleic acid

MDR, multidrug-resistant

PCR, Polymerase chain reaction

RNA, ribonucleic acid

STEC, Shiga-toxin-producing *E. coli*

μl, microliter

°C, degree Celsius.
